# Testing for COVID-19 cases in ASEAN

**DOI:** 10.5365/wpsar.2020.11.1.013

**Published:** 2020-11-13

**Authors:** David SQ Koh, Sheena XM Wong, Justin Wong

**Affiliations:** a Universiti Brunei Darussalam.; b Ministry of Health, Brunei Darussalam.

How many people actually have coronavirus disease 2019 (COVID-19)? Valid data describing the number and distribution of COVID-19 cases are critical for the design and implementation of containment strategies; however, timely and accurate measurement of disease incidence continues to pose challenges. (*1*) To obtain an accurate picture of the scale of the outbreak, we reviewed the count of cases and tests, as well as the testing rate and the proportion of positive tests, in Member States of the Association of South-eastern Asian Nations (ASEAN). ASEAN is a 10-member grouping of countries with a population of over 650 million people. (*2*) Cases of COVID-19 within ASEAN were reported first in Thailand and then Singapore in January 2020 and later in other ASEAN countries, with the first death outside China reported in the Philippines. To date, COVID-19 has affected all countries in the ASEAN region, with many imposing community quarantines and all restricting travel to varying degrees. (*3*)

No country knows the true number of people infected with COVID-19. All that can be ascertained is the status of those who have been tested. The total number of people who have tested positive – the number of confirmed cases – is not the total number of people who have been infected. The number of COVID-19 cases reported in a country is dependent on its surveillance sensitivity and laboratory testing capacity. The criteria for laboratory testing are also important because countries screen and test “suspect cases” based on clinical symptoms and a relevant epidemiological history. (*4*, *5*) It is likely that in some ASEAN countries, cases of COVID-19 may be undetected because of restrictive case definitions of suspect cases or limited testing capability to possible issues with case ascertainment in the early phases of ASEAN countries’COVID-19 responses, we reviewed information on testing and cases obtained from ministries of health and online news sources from March–April 2020 (**Table 1**). We provide an overview important testing indicators in ASEAN countries during this phase of the COVID-19 pandemic and discuss the utility of testing coverage, positivity rate and criteria for testing.

**Table 1:** Confirmed COVID-19 cases and laboratory tests performed in ASEAN countries (March–April 2020)

Country	Population (millions)	Confirmed cases		No. of individuals tested		Individuals tested per 100 000		Test positivity rate (%)		WHO transmission classification as of 30 Apr
		Early Mar	29 Apr	Early Mar	End Apr	Early Mar	End Apr	Early Mar	End Apr	
Singapore	**5.8**	117	15 641	1300^25F^	99 929^27A^	23	1772	9	16	Clusters of cases
Malaysia	**32**	55	5945	1000^25F^	154 203^29A^	3	489	5.5	4	Clusters of cases
Thailand	**69**	47	2947	3680^3M^	62 018^29A^	5	89	1.3	5	Clusters of cases
Viet Nam	**97**	16	270	N/A	261 004^29A^	0	273	N/A	0	Clusters of cases
Philippines	**109**	3	8212	N/A	88 869^28A^	0	83	N/A	9	Clusters of cases
Indonesia	**273**	2	9771	331^3M^	67 784^29A^	0	18	0.6	14	Community transmission
Cambodia	**16.7**	1	122	227^5M^	11 576^27A^	1	71	0.44	1	Sporadic cases
Myanmar	**54**	0	150	43^5M^	7718^29A^	0	14	0	2	Clusters of cases
Lao People's Democratic Republic	**7.2**	0	19	54^5M^	1796^27A^	1	25	0	1	Sporadic cases
Brunei Darussalam Darussalam	**0.43**	0	138	32^5M^	13 428^29A^	7	3130	0	1	Sporadic cases

^25F^ as of 25 February,^5M^ as of 5 March,^28A^ as of 28 April,^3M^ as of 3 March,^27A^ as of 27 April,^29A^ as of 29 April

*Source:* **Ministries of health; WHO Coronavirus Disease 2019 (COVID-19) Situation Report – 101, 30 Apr 2020.**

Note: All ASEAN countries require a positive polymerase chain reaction test for SARS-CoV-2 to confirm a case.

One indicator of the reliability of testing data is testing coverage, or the number of tests conducted per 100 000 population. Generally, we would expect that there are two reasons that countries with higher testing coverage have more reliable data on confirmed cases. First, a greater degree of testing provides us with a larger “sample” of people for whom disease status is known. Second, it may be the case that where the capacity for testing is low, tests may be reserved for particularly high-risk groups. Such rationing is one reason that those people tested may not be representative of the wider population. As observed in **Table 1**, all countries increased their testing coverage from March to April 2020, and marked heterogeneity exists across countries. We note that across all countries, the number of cases increased dramatically in March and April 2020. In addition to the expansion of testing for severe acute respiratory syndrome coronavirus 2 (SARS-CoV-2), the virus that causes COVID-19, this pattern of rapid case increase in some countries could also have been reflective of community transmission.

A second indicator we highlight in **Table 1** is the test positivity rate (TPR), defined as the number of confirmed COVID-19 cases per 100 tests. TPR is widely used by malaria surveillance programmes as one of several key indicators of temporal trends in malaria incidence. (*6*) For COVID-19, WHO interim guidance notes percent positive as an epidemiological factor to be used in risk assessments for countries. (*7*) We support that this indicator may be of utility with respect to the assessment of COVID-19 in ASEAN country contexts. At the beginning of the outbreak, when the COVID-19 caseload was low, a smaller number of tests was needed to accurately assess the spread of the virus. As the disease spreads, testing coverage needed to expand to provide a reliable picture of the true number of infected people. TPR can also be useful in determining whether or not an apparent increase in incidence is a result of better case detection, or a true increase. For example, if incidence is calculated using total population as a denominator and is found to be higher than usual, this could indicate a true increase in incidence. However, if the TPR shows a declining trend, it may suggest that this apparent increase is actually a result of better testing. (*8*) Here, we observe wide discrepancies across countries, with Viet Nam having a TPR of 0.1%, compared with Singapore having a TPR of 16%. A high TPR could be an indicator of underdetection, as tests may be reserved for those with a high probability of having the disease. Individuals who have the disease but are asymptomatic may not be detected. Broad surveillance strategies should encounter far more people who are not infected than people who are, so the TPR should be lower.

Based on our findings, we make three observations. First, some countries established additional surveillance by performing laboratory tests for SARS-CoV-2 for patients with pneumonia or selected community cases of influenza-like illness. (*9*)

To get a clearer sense of SARS-CoV-2 transmission intensity, countries need to broaden criteria for testing. Rather than testing solely based on restrictive suspect case definitions, population or clinic-based fever surveillance, along with testing suspect cases, would provide a more accurate understanding of SARS-CoV-2 transmission. This is particularly relevant in view of the varied and non-specific clinical presentation of COVID-19. Both random testing and targeted testing, particularly among individuals in high-risk settings such as health care, have merit and need to be conducted simultaneously.

Second, all cases reported by ASEAN countries in this data set were diagnosed based on viral detection by polymerase chain reaction tests, which cannot detect resolved infections, (*9*) and depending on testing criteria, may have missed asymptomatic or mild infections, because such people may not be tested. While acknowledging current limitations and variable test performance characteristics, we recommend large-scale seroprevalence studies as one additional measure to identify the best available estimate of disease burden and to compare with reports from existing surveillance systems. Longitudinal cohort studies that estimate seroprevalence can also assist in estimating trends in disease transmission over time.

Third, as a regional grouping, with significant volumes of travel and trade among Member States, ASEAN countries have a vested interest in assessing disease burden as they move towards de-escalation of travel restrictions and other restrictions on movement. To increase transparency and build trust, we suggest that countries report not only the number of confirmed cases in their country, but also the number of people tested or tests performed, and the criteria for such testing.
